# Virtual Reality Experience during Labour (VIREL); a qualitative study

**DOI:** 10.1186/s12884-023-05432-9

**Published:** 2023-04-24

**Authors:** A. Musters, A. S. Vandevenne, A. Franx, M. M. L. H. Wassen

**Affiliations:** 1 Department of Obstetrics and Gynaecology, Zuyderland Medical Centre, P.O. Box 4446, Heerlen, 6401 CX The Netherlands; 2 Maastricht University, P.O. Box 616, Maastricht, 6200 MD The Netherlands; 3 Department of Obstetrics and Gynaecology, Erasmus Medical Centre, P.O. Box 2060, Rotterdam, 3000 CB The Netherlands

**Keywords:** Virtual reality, Labour, Pain, Analgesia, Obstetric care

## Abstract

**Background:**

There is increasing evidence that virtual reality (VR) is effective in the reduction of labour pain. The implementation of alternative methods like VR to reduce labour pain can contribute to reduce patient request for pharmacological pain management methods and associated side effects. The aim of this study is to examine women’s experiences, preferences and satisfaction in regard to the use of VR during labour.

**Methods:**

A qualitative interview study was conducted in a non-university teaching hospital in The Netherlands. Two VR applications, respectively a guided meditation and an interactive game were tested in eligible women with a singleton pregnancy, scheduled for induction of labour. For the primary outcome, patients’ VR experience and application preference (meditation vs. game) were examined using a post-intervention questionnaire and a semi-structured interview. Three categories (with sub-categories) were used to guide interviews: “The VR experience”, “Pain reduction”, and “Usability of the VR application”. Labour pain before and directly after VR was evaluated using the NRS score.

**Results:**

Twenty-four women, of whom 14 were nulliparous and ten multiparous, were included and 12 of these women participated in semi-structured interviews. Using within-subject paired t-test comparisons, compared to pain pre-VR, patients reported a highly significant 26% decrease in mean NRS scores during VR meditation (pain pre-VR = 6.71 + − 1.65 vs. pain after VR = 4.96 + − 2.01) [*p* < 0.001]. Compared to pain before VR game, patients also reported a highly significant 19% decrease in mean NRS scores during VR game (pain before VR game = 6.89 + − 1.88 vs. pain after VR game = 5.61 + − 2.23) [*p* = 0.001].

**Conclusion:**

All women were highly satisfied with VR use during labour. Patients reported a highly significant reduction in pain during the interactive VR game and during meditation, patients preferred guided meditation. These results can contribute to the development of a potential promising new non-pharmacological tool to reduce labour pain.

**Trial registration:**

ClinicalTrials.gov Identifier: NCT04858984, date of registration: 26/04/2021 (retrospectively registered).

## Background

Epidural Analgesia (EA) has been regarded as the most effective method of labour analgesia [1]. There is a wide variation internationally in the use of intrapartum pharmacological analgesia, varying from 22.3% in The Netherlands to 83.1% in Sweden in nulliparous women [2]. The rates of EA during labour in nulliparous women varies from 19.4% in England to 83.4% in Finland [2]. Pharmacological analgesia methods are known to have adverse side effects, such as nausea, vomiting and drowsiness in parental opioids (intramuscular and intravenous drugs like patient-controlled analgesia); pruritus, nausea, desaturation, respiratory depression and apnea in patient-controlled remifentanil analgesia; and maternal fever, maternal hypotension and urinary retention in EA. Furthermore, routine EA is likely resulting in more operative deliveries [3–6]. Considering these possible adverse effects, it is worth exploring alternative methods for labour pain relief.

Virtual reality (VR) is increasingly used in healthcare, and makes use of the principle of distraction [7]. Pain perception is strongly affected by psychological factors [8, 9]. The perception of pain is thought to be (partly) related to the amount of attention that is given to pain stimuli [10, 11]. A systematic review and meta-analysis showed interactive VR to reduce acute pain during dressing changes in adults with burns or cut wounds [12]. The VRAIL pilot study, a preliminary randomized controlled trial of 27 women, showed a significant decrease in pain and anxiety during labour in the VR-group [13]. A recently published review including nine studies related to VR during pregnancy and delivery, discussed the potential of VR as an effective method for pain and anxiety [14].

VR is inexpensive and can be used as a safe, non-invasive, analgesic method, without risks of drug addiction and minimal side effects [15, 16]. Therefore, VR has tremendous potential as a safe alternative or adjunct to existing pharmacological analgesia during labour. However, implementation of VR in obstetric care requires insights in labouring women’s preferences and pain reduction perceived by women. This study aims to examine the experience, preference, and satisfaction of VR in women during labour.

## Methods

### Study design and participants

The study was set up as a single centre, qualitative study in a non-university teaching hospital in The Netherlands. Women were included between July 2020 and January 2021. This study was approved by the Medical Review Ethics Committee and registered as a clinical trial trial (ClinicalTrials.gov Identifier: NCT04858984, date of registration: 26/04/2021).

Inclusion criteria were women of 18 years or older, native Dutch speaker, pregnant of a singleton in cephalic position, intention to deliver vaginally, and scheduled for induction of labour. Exclusion criteria were woman with chronic pain (persistent or recurrent pain > 3 months and not due to the pregnancy), chronic use of opioids, alcohol or drug abuse, known predisposition to motion or cyber-sickness, epileptic insults in previous history, claustrophobia, blindness, and severe hearing or vision deficits [16–18].

When scheduled for induction of labour women were given oral and written information about the study. Informed consent was obtained on the day of induction. The required number of patients for the qualitative data was determined by saturation: new patients were included until no new information was obtained. All collected data were stored in an electronic case report form (eCRF). This article has been written according to the consolidated criteria for reporting qualitative research (COREQ) [19].

### Intervention

Participants experienced an immersive guided meditation (VR meditation) and an interactive game experience (VR game) during labour. A portable, standalone VR headset called Oculus Go (Facebook Technologies, LLC. 1601 Willow Road, Menlo Park, CA 940250) with a head-mounted display with built in audio drivers was used. Disposable hygiene masks and a surgical cap were used as an underlay below the headset and headset was cleaned afterwards according to a standard hygiene protocol.

VR meditation consisted of a video of an exotic location guided by the sound of the waves and a calm English-speaking voice. VR game required women to use the controller to throw snowballs in order to catch presents and reach the next level. Patients were allowed to stop using the VR at any moment during the intervention.

When the participant was declared to be in labour, defined as having regular painful uterine contractions, cervical effacement and at least 3 cm dilatation, VR meditation was offered for 10 min [20]. Before and immediately after VR meditation, the patient was asked to fill out a Numeric Rating Scale (NRS) score for pain.

During the 30-min intermission after VR meditation the patient completed the post-intervention questionnaire regarding VR meditation. Subsequently, the VR game was offered for 10 min. Before and immediately after VR game the patient filled in the NRS score for pain, and the patient completed a post-intervention questionnaire regarding VR game. Additional use of VR was allowed by maternal request and noted in the eCRF. If a woman had a request for pharmacological analgesia this was offered according to the local protocol. Five days post-partum all participants who completed both VR interventions were contacted by telephone for an interview.

### Questionnaires and semi-structured interviews

The NRS score indicates the degree of pain on a scale from 0 to 10, where 0 means no pain and 10 is the worst pain imaginable [21]. In the post-intervention questionnaire the experience of both VR interventions were asked.

Three categories (with sub-categories) were used to guide interviews: “The VR experience”, “Pain reduction”, and “Usability of the VR application”. The audiotaped interviews were conducted by AM and AV and lasted approximately 20 min. The interviews were semi-structured as they were based on a topic list (Table 1), and used to gain information about the preferences and experience of the VR applications during labour. As the interviews proceed, questions are adapted depending on the answers given to gain more depth and to verify whether the opinions agree between the participants.

**Table 1:** Topic list semi-structured interviews (supporting information)

Panel: Theme categories and clusters of semi-structured interviews	
1. VR experience	
1.1 General experience	
1.2 VR meditation experience	
1.3 VR game experience	
1.4 Preference	
1.5 Side effects	
1.6 Reusing VR	
1.7 Recommendation to other women	
1.8 Improvements	
2. Pain reduction	
2.1 Pain intensity	
2.2 Pain perception	
2.3 Distraction	
3. Usability VR application	
3.1 VR application	
3.2 Comfort of VR glasses	

### Data analysis

The baseline characteristics and the NRS score data were analysed using SPSS (version 27). A within-subject paired t-test was used to analyse the difference in NRS score pre- and post-VR intervention. All interviews were transcribed verbatim. The interview transcripts were analysed by AM and AV. ATLAS.ti (version 8) was used by the investigators to code, organize and manage the data to facilitate data interpretation. MW read and coded 20% of the transcripts again, as a validity check. Findings were compared and discussed by AM, AV, and MW until consensus on codes was achieved. A thematic analysis method was used for analysing the qualitative data.

Quotes were translated from Dutch to English by AM and AV, AF translated the English quotes back into Dutch sentences to make sure that the translation was substantively correct.

## Results

**Fig. 1:**
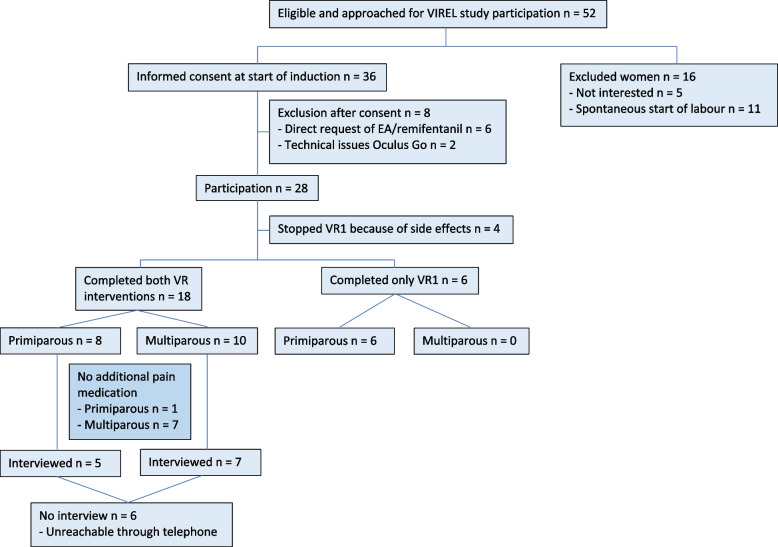
Study algorithm and sample characteristics

A total of 52 women were approached to participate and 36 women gave informed consent (Fig. 1). Eight women (22%) were excluded: six immediately requested pharmacological analgesia and two participants encountered technical issues with the VR glasses. Of the 28 women who used VR, four (14,3%) stopped immediately after starting: three women because of cyber-sickness and one woman felt uncomfortable wearing the VR glasses.

Both VR interventions were completed by 18 of 24 women (75%), eight (44.4%) were primiparous (i.e., had previously given birth to one child) and ten (55%) were multiparous (i.e. had previously given birth to more than one child). Eight (44.4%) of the 18 women who completed both VR interventions requested no additional pain medication during labour of which seven (87.5%) were multiparous. Six of 24 (25%) women completed only VR meditation of which five (83.3%) requested pharmacological pain medication after VR meditation and one participant had to stop because of a technical issue with the Oculus Go. Additional time in VR during labour was requested by ten (41.7%) women.

Our quota for data collection concerning experience, preference and satisfaction of VR use was reached after 12 semi-structured interviews.

Baseline characteristics of the study group were reported in Table 2 and of the interviewees in Table 3. As expected, multiparous women were significantly older than nulliparous women. All women were Caucasian. By one woman out of 28 participants, induction was started by artificial rupture of membranes, all other inductions were started by using a foley balloon catheter. The balloon puts pressure on the cervix to cause it to dilate to induce labor.

**Table 2:** Baseline characteristics of participants

	All women ***n*** = 28	Primiparous women ***n*** = 16	Multiparous women ***n*** = 12	***P***-value (α = 0·05)
Age, mean ± SD, years	30·82 ± 3·86	28·88 ± 3·78	33·42 ± 2·07	0·001
BMI before pregnancy, mean ± SD, kg/m2	26·93 ± 5·90	27·03 ± 5·37	26·78 ± 6·79	0·915
Gestational age, mean ± SD, weeks	39·21 ± 1·50	39·18 ± 1·63	39·25 ± 1·36	0·907

**Table 3:** Data of interviewees

	Parity	Age, years	Gestational age, weeks + days	Profession	Repeated use of VR	Additional pharmacological analgesia after VR	VR-preference
VIREL-1	Multiparous	35	41 + 2	Nurse	VR1	No	VR1
VIREL-2	Multiparous	33	39 + 0	Doctor	No	Yes, EA	VR1
VIREL-7	Primiparous	29	41 + 4	Social worker	No	Yes, remifentanil	VR1
VIREL-10	Multiparous	36	38 + 1	Disability support worker	VR1	No	VR1
VIREL-12	Multiparous	33	38 + 2	Administrative assistant	VR1 + VR2	No	VR1 + VR2
VIREL-14	Primiparous	32	39 + 0	Childcare worker	VR1	Yes, remifentanil	VR1
VIREL-16	Multiparous	31	38 + 0	Childcare worker	No	No	VR2
VIREL-19	Primiparous	33	38 + 6	Nurse	No	Yes, EA	VR1
VIREL-20	Multiparous	35	38 + 1	Housewife, completed general secondary school	VR1	Yes, remifentanil	VR1
VIREL-23	Primiparous	25	38 + 1	Nurse	No	Yes, EA	VR2
VIREL-25	Primiparous	28	38 + 1	Estate agent	No	Yes, EA	VR1
VIREL-27	Multiparous	30	40 + 1	Entrepreneur	No	No	VR2

*VR1* VR meditation, *VR 2* VR game

Data is presented within the three categories and subcategories that guided the semi-structured interviews: (1) the VR experience, (2) the pain reduction, and (3) the usability of the VR-application.

### Category 1: the VR experience

#### General experience

Overall, all interviewees experienced positive effects when using VR.

“Yes, it surely was a positive experience... A good way to detach myself from labour pain… I think VR is especially useful to relax in between the contraction” (VIREL-20)

“It was great. I think I had a dilation of four centimetres when I used the VR for the first time and stopped using VR at eight centimetres.” (VIREL-1)

Additionally, women lost perception of the external environment while using the guided meditation VR.

“I prefer the guided meditation for several reasons: the relaxing sound of the waves, the voice guidance, and the perception of being in a pleasant environment instead of being in a hospital.” (VIREL-25)

All women describe VR as a good distraction method during labour.

“You mentioned talking to my partner and your telephone ringing while I was using VR, but I did not remember hearing any of it.” (VIREL-2)

“I was so distracted by the beach environment and the meditation voice, that I was less aware of the pain I was in.” (VIREL-20)

#### VR meditation experience

Four women reported that the guided meditation VR helped them with their breathing technique.

“It helped me to control my breathing during a contraction” (VIREL-25)

The guided meditation VR provides a continuous support, which was mentioned as positive by multiple women. It enhanced their self confidence in bearing the labour pain.

“The continuous support of the VR voice was pleasant…It helped me cope with the pain” (VIREL-2)

The beautiful wide view and tranquillity was highlighted by women regarding the VR images, but a preference for less voice guidance was also expressed.

“It is important that the environment is interesting and has a wide view.” (VIREL-2)

“I liked the voice guidance at some moments, but not the entire time. More moments of just music and nature sounds would be nice.” (VIREL-23)

#### VR game experience

All women reported the importance of relaxation during labour and stated that an interactive game was not suitable, because during the game they continuously tightened their muscles.

“I could not relax during the game which caused no reduction in pain.” (VIREL-7)

At some point during labour the experienced degree of pain was too high to focus on playing a game, which seems to make the interactive game less suitable during labour.

“The game could be really fun, but from my perspective it is not suitable during labour. I could not focus on the game.” (VIREL-1)

“I would prefer playing a game when I am in less pain, because now I could not concentrate on it.” (VIREL-2)

### Preference

Eight women preferred VR meditation, three women liked both VR interventions, and one woman reported no additional value of VR. She preferred her partner to help her cope with labour pain.

Eleven women described VR as helpful for coping with labour pain during the first stages of dilation, but when contractions became too severe and they felt lots of pressure, or the urge to push, they were unable to handle VR glasses any longer. Six out of the seven women who used no additional pain medication stopped using VR just before the second stage of labour.

“… but when the contraction and the labour pain got really intense, the beach was not comforting me anymore.” (VIREL-10)

“When I felt like pushing the VR glasses were not comfortable anymore.” (VIREL-1)

Nine women stated that they preferred their own native language during the guided meditation instead of a non-native language.

“Listening to a non-native language requires more energy.” (VIREL-10)

Multiple women mentioned the passive way of distraction as an advantages of VR mindfulness. Whereas the VR game was difficult to play during labour.

“The advantage of the guided meditation is that you do not need to actively participate in experience.” (VIREL-2)

#### Side effects

None of the women who completed both VR interventions reported side-effects.

#### Reusing VR

Except VIREL-16 all interviewees would use VR during a subsequent labour.

“I would definitely use the VR glasses again during a next delivery. I would like to use the VR glasses even longer to pass the time.” (VIREL-12)

#### Recommendation to other women

All women would recommend using VR during labour to cope with their contractions.

“I surely would, if it is not working for you, you can immediately stop using VR.” (VIREL-10)

“It is worth trying… Everyone has to experience it for themselves.” (VIREL-27)

#### Improvements

In this study, the intervention lasted 10 min. Seven women, however reported that a prolonged VR session without any intermission could have a better effect on long lasting pain reduction during labour. Time seemed to be going faster while using VR.

“Ten minutes is very short; I think using VR for 30 minutes or maybe an hour without intermission would be better.” (VIREL-2)

Regarding to the intention of longer VR use women would prefer different nature environments or at least a more entertaining environment to keep their attention.

“Although I love the beach, a change in different relaxing environments during a longer period of VR use would be nice.” (VIREL-14)

The importance of support of midwifes, nurses, and the partner to make VR successful was also mentioned by two women.

“If the midwife would have put more effort into convincing me to try the VR meditation of VR game again, I might have used VR another time… The support and persuasiveness of the caregivers regarding VR partly determines the success of VR.” (VIREL-2)

It seems to be important that the guided meditation is specifically developed for use during labour and that the voice guidance helps women cope with their contractions. Women reported they needed positive vocal support especially during the most painful contractions.

“When the labour pain got worse, I needed more coaching guidance and lots of positive mental support.” (VIREL-25)

“When experiencing severe labour pain, I needed a voice to help me cope with the contraction. Unfortunately, it was not possible with this application.” (VIREL-1)

Overall, partners were not bothered by their wife using VR. Although some partners stated they would like to see what she is experiencing on an iPad.

### Category 2: pain reduction

#### Pain intensity

All women reported a reduction in labour pain.

One woman felt no pain at all when using VR mindfulness.

“At the beginning I did not feel my contractions at all when using the guided meditation, after taking off the VR glasses I could not tell how many contractions I had in the last ten minutes.” (VIREL-1)

Eleven women describe their contractions as less intense and less painful when using VR.

“It reduced the pain to a level that makes it bearable.” (VIREL-25)

“… the contractions felt shorter and less intense” (VIREL-19)

Women described VR as an effective method to reduce pain, especially during the first stage of the active labour phase VR.

“At the end, my contractions were too painful to concentrate on the VR environment and could I not experience the relaxed feeling I experienced before.” (VIREL-25)

#### Pain perception

Women described VR meditation as a relaxing experience; they were more focused on the nature environment, the soothing voice and their breathing. This caused enough distraction to reduce the degree of experienced pain. Eleven participants experienced positive effects regarding to their pain perception and the tension in their body.

“Without VR I felt strong contractions and a lot of pelvic pressure, but when using VR, the perception of the contractions was less.” (VIREL-10)

#### Distraction

The distractive component of VR reduces the amount of experienced pain.

“Because of the distractive component of the VR I was not focused on my pain, but I still felt it.” (VIREL-27)

“The contractions were less intense because I was distracted by hearing the soothing voice.” (VIREL-14)

In the first stages of experiencing labour pain, a change in environment seemed to be important to keep a woman’s attention. In the later, more severe, stages of labour pain a change in environment was less important, at that point the vocal support during a contraction was more meaningful. VIREL-1 used the meditation VR the first time when having 4 cm dilatation and regularly contractions, the second time she used the meditation VR she had already 7 cm dilatation, 5 contractions every 10 min and she felt a lot of pressure.

“The first time I got bored after a couple of minutes seeing the same beach environment… The second time using the meditation VR, I did not pay much attention to the surrounding, so the change of environment was less important.” (VIREL-1)

“When the contractions became really severe, I was mainly focussed on the voice guidance.” (VIREL-10)

### Category 3: the usability of the VR-application

All women mentioned that it is important to keep the VR application as simple as possible regarding to the usability. All women were positive about the comfort of the VR glasses. Three women noted that it would be nice to use the VR glasses at least once before experiencing labour pain.

“It would be nice to try the VR glasses before being in labour, so you feel more comfortable using them when in pain...” (VIREL-16)

Verification of the qualitative data was done by comparison with the NRS scores.

The mean NRS scores before starting respectively VR meditation and VR game were 6·71 (SD1·65) and 6·89 (SD1·88) respectively (Table 4). The mean NRS scores after VR meditation and after VR game were 4·96 (SD2·01) and 5·61 (SD2·23) respectively (Table 4).

**Table 4:** NRS scores before and after VR intervention, paired t-test

	NRS before VR (mean ± SD)	NRS after VR (mean ± SD)	***P***-value (α = 0·05)
VR1, *n* = 24	6·71 ± 1·65	4·96 ± 2·01	0·000
Primiparous, *n* = 14	7·29 ± 1·27	5·64 ± 1·60	0·002
Multiparous, *n* = 10	5·90 ± 1·85	4·00 ± 2·21	0·010
VR2, *n* = 18	6·89 ± 1·88	5·61 ± 2·23	0·001
Primiparous, *n* = 8	7·50 ± 1·51	6·25 ± 1·98	0·026
Multiparous, *n* = 10	6·40 ± 2·07	5·10 ± 2·38	0·017

*VR1* VR meditation, *VR 2* VR game

The reported decrease in NRS score was 1·8 after VR meditation and 1·2 after VR game (Table 4).

## Discussion

### Summary of findings

All women were highly satisfied with VR use during labour and experienced less pain. Women preferred the guided meditation. In particular, multiparous women benefited from VR use. The significant difference in NRS score before and after the use of VR for both VR mindfulness and the interactive VR game supports the potential effectiveness of this innovative technique during labour.

When evaluating all participants, the guided meditation seems to be preferred during labour in comparison to the interactive game. This is supported by the qualitative and quantitative data of this study.

We evaluated suggestions for improvement made by the interviewees during the early and late dilatation stage of labour. During the latent phase and the early dilatation stage of labour (defined as regular painful contractions and a cervical dilatation of 3-5 cm) women preferred more distraction such as different nature environments, a more realistic image of the nature surrounding, and a meditative voice guidance. During the late dilatation stage (defined as a cervical dilatation 6-10 cm), when contractions are more painful, women preferred coaching, less distraction, and calm music or soothing nature sounds in between the contractions. Emotional support such as praising for endurance, positive words and psychical support like breathing techniques helped women to cope with labour pain [22]. Coaching and emotional support will be incorporated in the development of a future VR labour application.

Several women recommended that using VR during the weeks leading up to childbirth would be helpful to reduce anxiety before labour and prepare for labour by repetitive doing a meditation and practice the breathing exercises. Previous studies show the positive effect of the use of VR during pregnancy and delivery regarding anxiety- and pain management [14].

### Strengths and limitations

The strength of this study is that this is the first qualitative evidence of the satisfaction and preference of the use of VR during labour. These results can contribute to the development of a new VR tool and innovative techniques to reduce labour pain.

A limitation of this study was that, although the pain ratings were collected in person during childbirth, the post-treatment followup interviews were conducted by telephone, as the Covid-19 lockdown did not allow face-to-face interviews. Because of this, we may have missed nonverbal cues. However, each time an interviewee gave feedback, we summarized and repeated the feedback to verify that we understood the message correctly. Six interviews were not conducted because the patient could not be reached by telephone. Another limitation is that VR meditation always preceded interactive VR game. In the future, treatment order should be randomised. We only included women who were scheduled for an induction of labour. Literature suggests the active phase of labour in primiparous women is longer in induced labour than in spontaneous onset, this will lead to more exhaustion and a higher request for pharmacological analgesia in patients whose labor is induced (as in all patients in the current study) [23]. Therefore, we suspect that VR may be more effective in women who have a spontaneous onset of labour when looking at the effect of VR and additional request for pharmacological analgesia. On the other hand, VR seemed to have a positive effect on coping with labour pain, which could help make an induction more bearable and increase birth satisfaction.

### Interpretation

The guided meditation provides the experience of continuous support during labour without causing side effects.

There were no statistically significant differences in pain measured by NRS scores between primiparous and multiparous women. However, multiparous women requested pharmacological analgesia less often. This is possibly related to a shorter duration of the active stage of labour in multiparous women [23]. Therefore, multiparous women stayed motivated to use VR.

Previous studies have demonstrated that VR is an effective tool to manage pain and distress in several medical procedures, such as wound care, dental procedures and dialysis [24]. The effect of VR on this short acute pain moments cannot be compared with long term pain management during labour. A RCT with 40 nulliparous women, of whom 21 received 30 min VR during labour, showed a significant reduction of pain in the VR group [25].

The interviewees reported the importance of support by midwives, nurses and the partner to make VR a successful alternative to pharmacological analgesia. It is possible that the positive effect of VR can be partly explained by offering continuous support. Continuous support during childbirth by a midwife, partner or doula is highly appreciated by women in labour and results in better birth experiences and a decrease in analgesia use, shorter duration of labour, increase in spontaneous vaginal birth and improved 5-min Apgar score [22, 26, 27].

## Conclusion

This study demonstrates VR to be a potentially valuable non-pharmacological tool to help reduce labour pain. The interviewees in this study were highly satisfied with VR, and suggested improvements such as using VR for a longer period of time, and a VR application specially aimed for women in labour. These suggestions can contribute to developing a specific labour VR application and improve the effectiveness.

Randomised controlled trials have to be performed to assess the effect of VR on the request for pharmacological analgesia by women in labour before VR can be implemented as routine standard care. More research and development is recommended.

## Data Availability

The datasets used and analyzed during the current study are available from the corresponding author on reasonable request.

## Abbreviations

VR: Virtual reality
EA: Epidural analgesia
eCRF: Electronic case report form
NRS: Numeric Rating Scale

## References

[1] Anim-Somuah M, Smyth RM, Cyna AM, Cuthbert A (2018). Epidural versus non-epidural or no analgesia for pain management in labour. Cochrane Database Syst Rev.

[2] Seijmonsbergen-Schermers A, Akker T, Rydahl E, Beeckman K, Bogaerts A, Binfa L (2020). Variations in use of childbirth interventions in 13 high-income countries: a multinational cross-sectional study. PLoS Med.

[3] Jones L, Othman M, Dowswell T, Alfirevic Z, Gates S, Newburn M, et al. Pain management for women in labour: an overview of systematic reviews. Cochrane Database Syst Rev. 2012;2012(3):CD009234. 10.1002/14651858.CD009234.pub2. 10.1002/14651858.CD009234.pub2 PMC7132546 22419342

[4] Wassen MM, Zuijlen J, Roumen FJ, Smits LJ, Marcus MA, Nijhuis JG (2011). Early versus late epidural analgesia and risk of instrumental delivery in nulliparous women: a systematic review. BJOG.

[5] Smith LA, Burns E, Cuthbert A (2018). Parenteral opioids for maternal pain management in labour. Cochrane Database Syst Rev.

[6] Van de Velde M (2015). Patient-controlled intravenous analgesia remifentanil for labor analgesia: time to stop, think and reconsider. Curr Opin Anaesthesiol.

[7] Indovina P, Barone D, Gallo L, Chirico A, De Pietro G, Giordano A (2018). Virtual reality as a distraction intervention to relieve pain and distress during medical procedures: a comprehensive literature review. Clin J Pain.

[8] McCaul KD, Malott JM (1984). Distraction and coping with pain. Psychol Bull.

[9] Katz J, Rosenbloom BN (2015). The golden anniversary of Melzack and Wall’s gate control theory of pain: celebrating 50 years of pain research and management. Pain Res Manag.

[10] Botella CPA, Banos R, Quero S, Breton-Lopez J (2008). Virtual reality in the treatment of pain. J Cyber Ther Rehabil.

[11] Guétin S, Brun L, Mériadec C, Camus E, Deniaud M, Thayer J, et al. A smartphone-based music intervention to reduce pain and anxiety in women before or during labor. Eur J Integr Med. 2018; in press.

[12] Mallari B, Spaeth EK, Goh H, Boyd BS (2019). Virtual reality as an analgesic for acute and chronic pain in adults: a systematic review and meta-analysis. J Pain Res.

[13] Frey DP, Bauer ME, Bell CL, Low LK, Hassett AL, Cassidy RB (2019). Virtual reality analgesia in labor: the VRAIL pilot study-a preliminary randomized controlled trial suggesting benefit of immersive virtual reality analgesia in unmedicated laboring women. Anesth Analg.

[14] Hajesmaeel-Gohari S, Sarpourian F, Shafiei E (2021). Virtual reality applications to assist pregnant women: a scoping review. BMC Pregnancy Childbirth.

[15] JahaniShoorab N, Ebrahimzadeh Zagami S, Nahvi A, Mazluom SR, Golmakani N, Talebi M (2015). The effect of virtual reality on pain in primiparity women during episiotomy repair: a randomize clinical trial. Iran J Med Sci.

[16] Li A, Montano Z, Chen VJ, Gold JI (2011). Virtual reality and pain management: current trends and future directions. Pain Manag.

[17] Treede RD, Rief W, Barke A, Aziz Q, Bennett MI, Benoliel R (2015). A classification of chronic pain for ICD-11. Pain.

[18] LaViola J (2000). A discussion of cybersickness in virtual environments. ACM SIGCHI Bull.

[19] Tong A, Sainsbury P, Craig J (2008). Consolidated criteria for reporting qualitative research (COREQ): a 32-item checklist for interviews and focus groups. Int J Qual Health Care.

[20] Hanley G, Munro S, Greyson D, Gross M, Hundley V, Spiby H (2016). Diagnosing onset of labor: a systematic review of definitions in the research literature. BMC Pregnancy Childbirth.

[21] Bijur PE, Latimer CT, Gallagher EJ (2003). Validation of a verbally administered numerical rating scale of acute pain for use in the emergency department. Acad Emerg Med.

[22] Beigi NM, Broumandfar K, Bahadoran P, Abedi HA (2010). Women’s experience of pain during childbirth. Iran J Nurs Midwifery Res.

[23] Ostborg TB, Romundstad PR, Eggebo TM (2017). Duration of the active phase of labor in spontaneous and induced labors. Acta Obstet Gynecol Scand.

[24] Wiederhold MD, Gao K, Wiederhold BK (2014). Clinical use of virtual reality distraction system to reduce anxiety and pain in dental procedures. Cyberpsychol Behav Soc Netw.

[25] Wong MS, Spiegel BMR, Gregory KD. Virtual reality reduces pain in laboring women: a randomized controlled trial. Am J Perinatol. 2020. 10.1016/j.ajog.2019.11.055. 10.1055/s-0040-1708851 32485759

[26] Lunda P, Minnie CS, Benade P (2018). Women’s experiences of continuous support during childbirth: a meta-synthesis. BMC Pregnancy Childbirth.

[27] Bohren MA, Hofmeyr GJ, Sakala C, Fukuzawa RK, Cuthbert A (2017). Continuous support for women during childbirth. Cochrane Database Syst Rev.

